# Concomitant Cervical Spondylotic Myelopathy and Transverse Myelitis Managed With C4-C6 Anterior Cervical Discectomy and Fusion

**DOI:** 10.7759/cureus.113244

**Published:** 2026-07-23

**Authors:** Michael Bridges, Ali J Hamade, Hussein Gharib, Nilesh M Patel

**Affiliations:** 1 Orthopedic Surgery, Beaumont Farmington Hills, Farmington Hills, USA; 2 Orthopedic Surgery, Corewell Health Farmington Hills, Farmington Hills, USA; 3 Orthopedic Surgery, Central Michigan University, Mount Pleasant, USA; 4 Orthopedic Surgery, Beaumont Health, Dearborn, USA

**Keywords:** anterior cervical discectomy and fusion, cervical myelopathy, degenerative cervical myelopathy, inflammatory myelopathy, mjoa, spinal cord compression, transverse myelitis

## Abstract

Cervical spondylotic myelopathy (CSM) and transverse myelitis (TM) may present with overlapping neurologic deficits, despite requiring distinct therapeutic approaches. We report the case of a 50-year-old female with progressive cervical myelopathy, severe C5-C6 stenosis, and a long-segment cervical cord signal abnormality extending beyond the level of maximal compression. Contrast-enhanced MRI revealed expansile intramedullary T2 hyperintensity with patchy enhancement, raising suspicion for TM. Extensive neurologic workup, including brain MRI, cerebrospinal fluid (CSF) analysis, oligoclonal bands, cytology, and infectious studies, was negative. Following multidisciplinary evaluation, the patient received intravenous methylprednisolone, followed by C4-C6 anterior cervical discectomy and fusion (ACDF). Neurologic function improved substantially, with the modified Japanese Orthopaedic Association (mJOA) score improving from 10 preoperatively to 16 at one year. This report highlights the importance of recognizing concomitant inflammatory and compressive spinal cord pathology and demonstrates favorable outcomes following a coordinated medical and surgical management approach.

## Introduction

Degenerative cervical myelopathy (DCM), often termed cervical spondylotic myelopathy (CSM) when cervical spondylosis is the primary etiology, is a common cause of progressive spinal cord dysfunction in adults. Epidemiologic estimates suggest a minimum incidence and prevalence of 41 and 605 per million, respectively, in North America [1]. The disorder predominantly affects older adults; in one observational series, the mean age at diagnosis was 63.8 years, with a male-to-female ratio of approximately 2.7:1 [2]. Common manifestations include gait imbalance, hand clumsiness, weakness, sensory disturbances, and upper motor neuron signs [1,3]. Transverse myelitis (TM) is a rare inflammatory spinal cord disorder that can affect individuals of both sexes and all age groups. An early population-based study estimated an annual incidence of 1.34 per million [4,5]. TM may present with weakness, sensory changes, gait dysfunction, and autonomic symptoms, resulting in substantial clinical overlap with CSM [4,6]. Distinguishing compressive myelopathy from inflammatory myelitis therefore requires careful correlation of symptom onset and progression, clinical examination, imaging, and laboratory evaluation.

The coexistence of CSM and TM presents a diagnostic and therapeutic challenge. Mechanical compression may warrant decompression, whereas inflammatory myelitis requires neurologic evaluation and medical therapy. Studies investigating the relationship between cervical degeneration and inflammatory or demyelinating spinal cord disease have yielded conflicting findings. Zhou et al. proposed an association between cervical disc degeneration and cervical non-infectious inflammatory myelitis [7]. In patients with multiple sclerosis, Gratch et al. found that spinal cord segments with at least moderate cervical stenosis were associated with demyelinating lesions at the same level, whereas Alkrenawi et al. reported no association between the severity of cervical disc disease and demyelinating spinal cord lesions [8,9].

These conflicting data underscore the uncertainty regarding a causal relationship. The case presented here warrants documentation because the coexistence of inflammatory and compressive pathology can confound diagnosis, alter treatment sequencing, and delay neurologic recovery. Longitudinal clinical and imaging follow-up provides practical insights into multidisciplinary management. We report this case in accordance with CARE principles [10].

## Case presentation

In February 2025, a 50-year-old female presented to her primary care physician (PCP) with a one-week history of bilateral upper extremity numbness and paresthesias involving both arms and hands. She denied any history of trauma but reported a remote history of intermittent neck pain without prior intervention. She had no relevant medical comorbidities. One week after her initial presentation, she was referred to our office. At the spine clinic, she presented with worsening axial neck pain and chronic low back pain. Her neck pain was rated 8/10 on the visual analog scale (VAS) and described as sharp and stabbing. She also reported shooting pain down the right arm, soreness in the left forearm, and bilateral hand numbness. She reported worsening hand dexterity, difficulty with overhead activity, dropping objects, weak grip strength, and difficulty performing activities of daily living. She required a walker for ambulation. She also reported chronic low back pain with posterior thigh radiation and right foot dragging for two months before her presentation.

Neurologic examination demonstrated an ataxic and unsteady gait. Deep tendon reflexes were brisk at 3/4 in the brachioradialis, biceps, triceps, patellar, and Achilles tendons bilaterally. Hoffmann sign was positive on the right and negative on the left. Disequilibrium was noted with rapid hand movements, and clonus was absent. Sensation to light touch and pinprick was diminished bilaterally from C4 through T2. Motor strength was graded using the Medical Research Council (MRC) scale and demonstrated 4-/5 strength in the deltoids, biceps, triceps, wrist flexors, and wrist extensors, while grip strength was graded at 3/5 bilaterally. Lower extremity strength was 4/5 throughout except for right ankle dorsiflexion, which was 2/5. Mild thenar eminence atrophy was noted. Based on the progressive bilateral sensorimotor symptoms, gait dysfunction, hyperreflexia, and right Hoffmann sign, the initial differential diagnosis included compressive cervical myelopathy, inflammatory or demyelinating spinal cord disease, and other neurologic or autoimmune etiologies.

Cervical and lumbar MRI studies were ordered to evaluate the progressive cervical symptoms and assess for lumbar pathology that might explain the lower extremity findings. No cervical CT was obtained because the clinical presentation raised concern for spinal cord or neurologic pathology rather than isolated osseous disease; MRI was selected to evaluate spinal cord compression, intramedullary signal abnormalities, and possible demyelinating disease. The patient was placed in a rigid Aspen collar. Laboratory evaluation showed C-reactive protein (CRP) 0.5 mg/L (reference range: < 8.0 mg/L), erythrocyte sedimentation rate (ESR) 1 mm/hr (reference range: 0-18 mm/hr), negative rheumatoid factor (RF), and positive antinuclear antibody (ANA).

Initial cervical MRI without contrast demonstrated multilevel spondylosis with moderate C4-C5 and severe C5-C6 canal stenosis, as well as abnormal intramedullary T2 hyperintensity extending from C3-T1 with cord expansion at C5-C6 and the C6 level (Figures 1A-1B). Lumbar MRI demonstrated mild degenerative changes without high-grade canal stenosis or compressive pathology to explain her lower extremity symptoms (Figure 2). Because the signal abnormality extended beyond the level of maximal compression, additional diagnostic evaluation was pursued.

**Figure 1:**
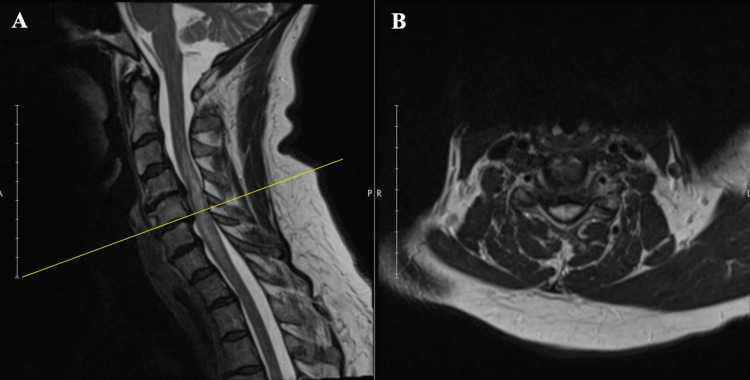
Initial preoperative cervical MRI without contrast Sagittal (A) and axial (B) T2-weighted images demonstrate multilevel degenerative cervical spondylosis with severe stenosis and long-segment intramedullary T2 hyperintensity extending beyond the level of maximal compression MRI: magnetic resonance imaging

**Figure 2:**
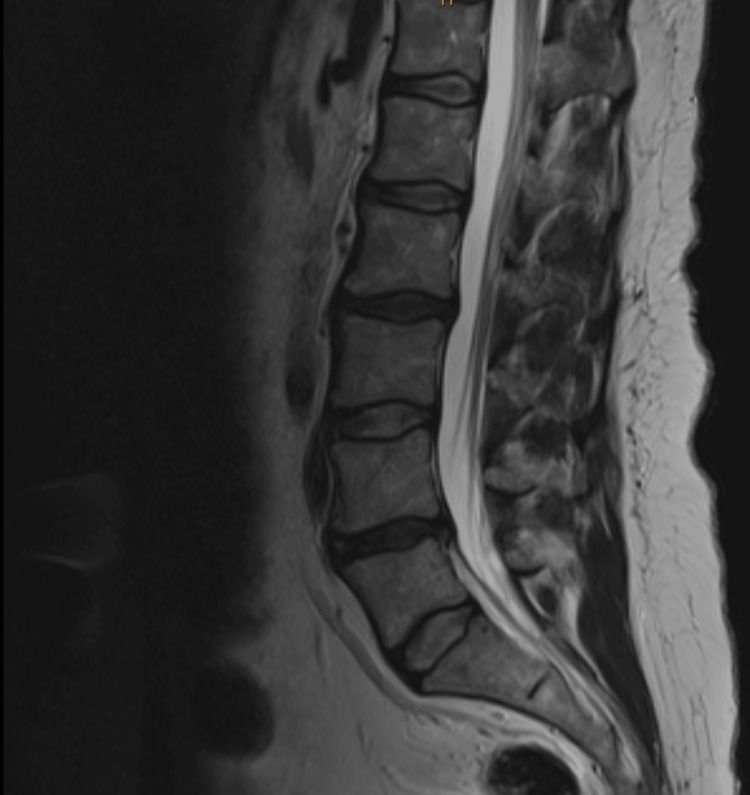
Preoperative lumbar MRI without contrast Mid-sagittal T2-weighted lumbar MRI demonstrating mild degenerative changes without significant central canal stenosis, neural element compression, or other lumbar pathology MRI: magnetic resonance imaging

Brain MRI showed no intracranial demyelinating disease, mass, or pathologic enhancement. Repeat cervical MRI with and without contrast demonstrated a slightly expansile central cord T2 hyperintensity from C4-C7 with mild patchy enhancement (Figures 3A-3D). The primary radiographic differential favored TM, with an intramedullary neoplasm considered less likely. Lumbar puncture was performed to exclude malignancy, multiple sclerosis, and infection. Cerebrospinal fluid (CSF) was colorless and clear, with a normal nucleated cell count, mildly elevated protein, elevated glucose, negative cytology, absent oligoclonal bands, and negative infectious testing (Table 1).

**Figure 3:**
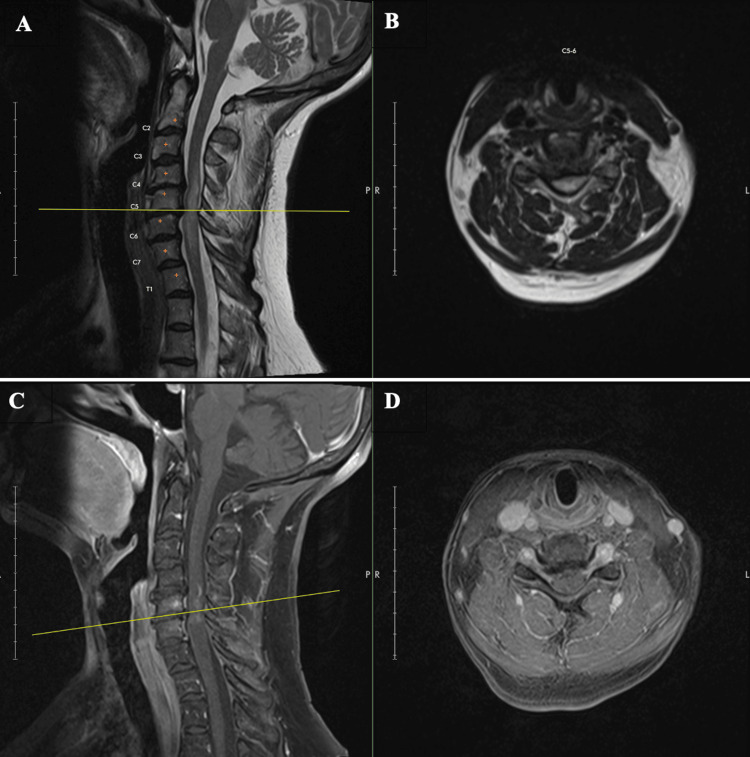
Repeat cervical preoperative MRI with and without contrast Sagittal and axial T2-weighted images (A-B) demonstrate persistent, slightly expansile central cord T2 hyperintensity centered from C4-C7. Post-contrast sagittal and axial post-contrast T1-weighted images (C-D) demonstrate mild patchy intramedullary enhancement, supporting an inflammatory myelitis pattern rather than isolated compressive myelopathy MRI: magnetic resonance imaging

**Table 1:** CSF results from lumbar puncture CSF: cerebrospinal fluid; HSV: herpes simplex virus; PCR: polymerase chain reaction; CMV: cytomegalovirus

CSF test	Result	Interpretation
Color/clarity	Colorless, clear	Normal
Xanthochromia	Absent	Normal
Total nucleated cells	1/mcL	Normal (reference ≤5/mcL)
Red blood cells	2/mcL	Elevated (reference <1/mcL)
Protein	55 mg/dL	Elevated (reference 15–45 mg/dL)
Glucose	102 mg/dL	Elevated (reference 50–80 mg/dL)
HSV-1/HSV-2 PCR	Not detected	Negative
CMV qualitative PCR	Not detected	Negative
Toxoplasma quantitative PCR	Not detected	Negative
Fungal culture	Negative	Negative
Acid-fast bacilli culture	Negative	Negative
Cytology	Negative for malignant cells	No evidence of malignancy
Oligoclonal bands	0 bands	Normal (reference 0–1 bands)

The patient was referred to neurology for further evaluation. Neurology diagnosed TM preoperatively and recommended high-dose corticosteroid therapy to decrease spinal cord inflammation, treat suspected demyelination, and minimize the risk of intraoperative secondary injury. The patient completed intravenous Solu-Medrol 1,000 mg daily for five days. Following completion of corticosteroid therapy and a negative workup for malignancy, multiple sclerosis, and infectious etiologies, she underwent C4-C6 anterior cervical discectomy and fusion (ACDF). At her final preoperative evaluation after completing corticosteroid therapy, objective examination demonstrated no change compared with her most recent preoperative clinic assessment. However, the patient reported subjective improvement in neurologic symptoms involving both upper extremities. Pre-positioning neuromonitoring was utilized because of the severe cervical stenosis and concomitant inflammatory spinal cord pathology. A standard left-sided anterior approach was performed without intraoperative complications or neuromonitoring changes.

At the first documented postoperative clinic visit, neck pain was 3/10 and described as a dull posterior neck ache consistent with expected surgical pain. Shooting pain down the right arm improved by 100%, left arm soreness improved by 50%, and burning dysesthesias in the hands and feet improved by 100%. Hand numbness continued to improve. Balance improved, although she still veered to the side while walking. She progressed from using a preoperative walker to primarily using a cane, while continuing to use a walker at home. A two-month postoperative cervical MRI with and without contrast showed improved canal patency on T2-weighted sequences and mild intramedullary enhancement consistent with persistent but slightly improved inflammatory myelitis on T1-weighted post-contrast images (Figures 4A-4D).

**Figure 4:**
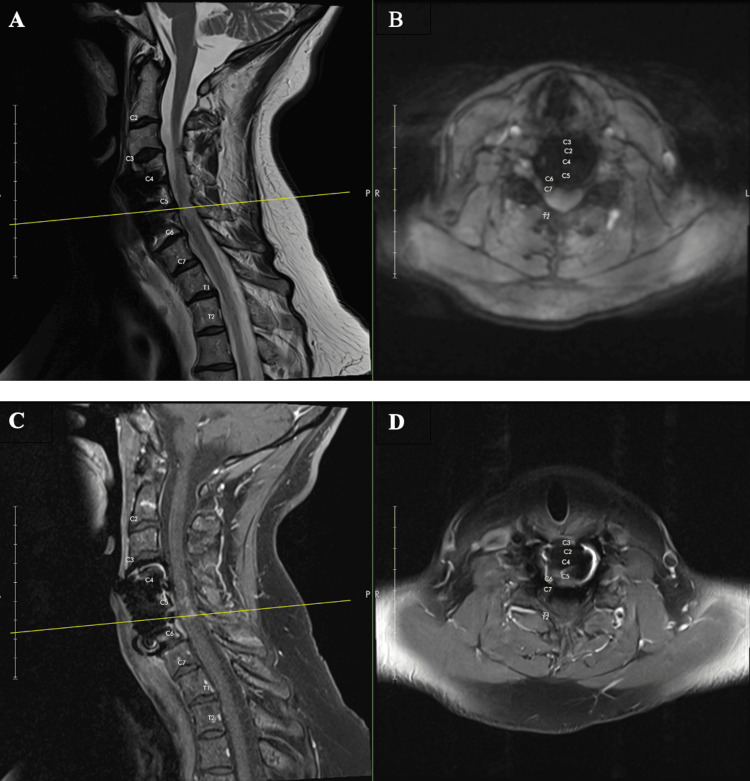
Two-month postoperative cervical MRI with and without contrast Sagittal and axial T2-weighted images (A-B) demonstrate postoperative changes after C4-C6 ACDF with improved canal patency and persistent but mildly decreased intramedullary signal abnormality. Sagittal and axial postcontrast T1-weighted images (C-D) demonstrate residual mild intramedullary enhancement consistent with persistent but minimally improved inflammatory myelitis MRI: magnetic resonance imaging; ACDF: anterior cervical discectomy and fusion

At the three-month follow-up, the patient reported approximately 85% overall improvement, although she continued to use a walker and reported residual arm and leg weakness with bilateral hand numbness and tingling. Her modified Japanese Orthopaedic Association (mJOA) score improved from 10 preoperatively to 13 at three months and 16 at one-year follow-up, representing a transition from severe to mild myelopathy based on established mJOA severity criteria [11]. At later follow-up, upper extremity sensation was documented as normal from C5-T1, and upper extremity strength was 5/5 in major muscle groups, although hyperreflexia and the right Hoffmann sign persisted. One-year postoperative MRI demonstrated mild signal abnormality on T2-weighted sequences and mild enhancement on T1-weighted post-contrast sequences, representing substantial improvement in both inflammatory and compressive findings compared with the prior imaging studies (Figures 5A-5D).

**Figure 5:**
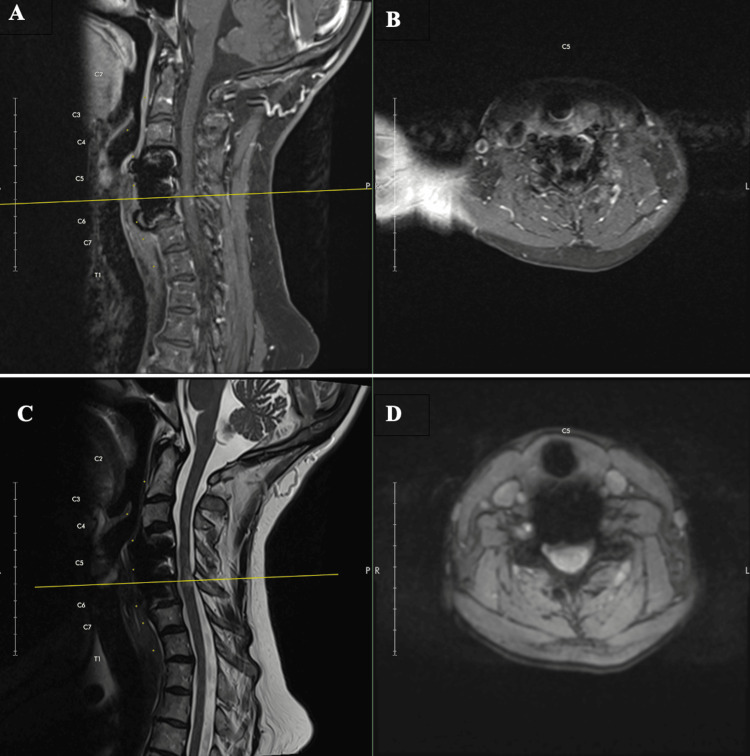
Follow-up postoperative cervical MRI with and without contrast Sagittal and axial T2-weighted pre-contrast images (A-B) demonstrate postoperative changes after C4-C6 ACDF with significantly decreased intramedullary signal abnormality. Sagittal and axial post-contrast T1-weighted images (C-D) demonstrate mild enhancement, demonstrating significant improvement and transverse myelitis when compared to previous films MRI: magnetic resonance imaging; ACDF: anterior cervical discectomy and fusion

## Discussion

This report illustrates the diagnostic complexity of overlapping CSM and TM. Both disorders can produce gait instability, limb weakness, hyperreflexia, sensory disturbances, and functional decline. Severe focal C5-C6 stenosis supported compressive myelopathy, whereas cord expansion and long-segment patchy enhancement extending beyond the level of maximal compression raised concern for inflammatory, demyelinating, infectious, or neoplastic disease. Established diagnostic criteria for TM require both exclusion of compression as the sole explanation and positive evidence of spinal cord inflammation; imaging findings alone are insufficient to confirm the diagnosis [4,6]. In this patient, the negative brain MRI, absent CSF oligoclonal bands, negative cytology, and negative infectious studies narrowed the differential and supported a concomitant inflammatory process because compressive myelopathy alone could not account for the full imaging phenotype.

Compressive myelopathy can itself produce long-segment T2 hyperintensity, cord expansion, and intramedullary enhancement, making comparison with prior reports essential. Kelley et al. described five patients referred for suspected neuromyelitis optica whose final diagnosis was symptomatic cervical stenosis; in each case, enhancement was localized to the level of maximal compression, symptoms responded poorly to corticosteroids, and surgical decompression produced clinical improvement [12]. The importance of recognizing compressive mimics is further illustrated by Flanagan et al., who in a 56-patient series reported longitudinal spindle-shaped T2 hyperintensity in all patients, cord enlargement in 79%, and a transverse pancake-like enhancement pattern in 73%; notably, 71% were initially diagnosed with neoplastic or inflammatory myelopathy, delaying decompression by a median of 11 months [13].

Intramedullary enhancement in compressive myelopathy is not rare: Ozawa et al. prospectively identified it in approximately 7% of 683 surgically treated patients [14]. The distinction from inflammatory disease is aided by lesion distribution; Sun et al. compared 27 patients with neuromyelitis optica spectrum disorder and 30 with long-segment DCM and found that compressive enhancement was typically limited to one level at or immediately below the site of maximal narrowing, whereas inflammatory lesions were more numerous and spanned longer segments [15]. Compared with these compressive patterns, the patchy enhancement from C4-C7, T2 signal from C3-T1, and subjective improvement after corticosteroids in our patient collectively favored an inflammatory component extending beyond the focal mechanical lesion. Nevertheless, because spondylotic myelopathy may also demonstrate enhancement, MRI findings alone cannot establish the presence of dual pathology.

Published cases further illustrate the consequences of attributing the entire presentation to a single mechanism. Wang and Newsome described two men with cervical spondylosis and enhancing intramedullary lesions who were treated for presumed inflammatory demyelinating disease but continued to decline; both ultimately underwent decompression, achieving improvement or stabilization despite persistent postoperative enhancement [16]. Similarly, Conway et al. reported a 55-year-old woman initially treated for a clinically isolated inflammatory syndrome whose persistent enhancement and dynamic compression on extension MRI ultimately prompted decompression, producing marked clinical and radiographic improvement [17].

These cases resemble ours in their overlapping symptoms and enhancing cord lesions but were ultimately interpreted as compressive mimics rather than simultaneous disease. The distinction in our patient lies in the combination of severe stenosis on neutral MRI, signal abnormality and patchy enhancement extending well beyond maximal compression, a negative neurologic workup, and subjective improvement after corticosteroids preceding decompression. Because both treatments preceded continued recovery, their individual contributions cannot be separated.

The treatment sequence was designed to address both plausible mechanisms concurrently rather than sequentially assuming one diagnosis. This approach is supported by Youssef et al., who in a series of 19 patients with demyelinating disorders and surgically treatable cervical degenerative disease reported significant postoperative neurologic improvement in more than 89% despite frequent delays in spine referral [18]. Our patient similarly improved after ACDF, with mJOA increasing from 10 to 16 and serial MRI showing improved canal patency and decreasing cord signal and enhancement. Neurology-directed corticosteroid therapy preceded decompression to address the suspected inflammatory component, while surgery addressed progressive mechanical compression in accordance with accepted DCM management principles [19,20]. The absence of intraoperative neuromonitoring changes and the improvement in pain, gait, strength, sensation, and mJOA score support the feasibility of coordinated treatment, although a single case cannot establish the optimal sequence or timing.

Evidence for a biologic association between degenerative stenosis and inflammatory or demyelinating cord disease remains inconsistent. Zhou et al. proposed that cervical disc degeneration may contribute to cervical non-infectious inflammatory myelitis [7]. Gratch et al. found that spinal segments with at least moderate stenosis were associated with multiple sclerosis (MS) lesions at the same level (odds ratio: 2.6), whereas Alkrenawi et al. found no relationship between disc-disease severity and demyelinating lesions in a separate MS cohort [8,9]. Differences in population, disease phenotype, imaging definitions, and segment-level methods may explain the conflicting results. Neither study establishes causality, and our case supports diagnostic vigilance rather than a causal inference.

This report is limited by its single-patient design, the absence of a disease-specific biomarker confirming TM, and the inability to determine the independent contributions of corticosteroid therapy and decompression. It also cannot define the optimal timing of steroids relative to surgery. Nevertheless, the case provides a clinically relevant example of multidisciplinary management for dual cervical spinal cord pathology. Strengths include detailed serial imaging, CSF evaluation, objective functional assessment with mJOA scores, documentation of the examination before and after corticosteroid therapy, and one-year follow-up.

## Conclusions

Concomitant CSM and TM represent a rare diagnostic and therapeutic challenge. Long-segment cord signal abnormality, cord expansion, or patchy enhancement extending beyond maximal stenosis should prompt evaluation for inflammatory, infectious, neoplastic, and demyelinating etiologies. In our patient, several converging findings supported dual pathology rather than a single unifying etiology. T2 signal abnormality spanning C3-T1 and patchy enhancement from C4-C7 extended well beyond the level of maximal compression at C5-C6, a distribution inconsistent with purely compressive myelopathy. The enhancement pattern was diffuse and patchy rather than the focal morphology characteristic of spondylotic disease. Comprehensive workup excluded MS, neuromyelitis optica, malignancy, and infectious myelitis, and independent neurologic evaluation supported the diagnosis of TM preoperatively. Subjective improvement following corticosteroid therapy further suggested an inflammatory component, as isolated compressive myelopathy would not be expected to respond to steroids. Serial postoperative imaging demonstrated improvement in canal patency and inflammatory cord signal along separate trajectories, consistent with two distinct but coexisting processes. No single finding was independently diagnostic; rather, the convergence of imaging distribution, enhancement morphology, negative biomarker workup, corticosteroid response, and longitudinal imaging evolution collectively supported the diagnosis. Preoperative Solu-Medrol therapy followed by C4-C6 ACDF led to substantial clinical improvement, with the mJOA score improving from 10 to 16 and serial MRI demonstrating improved decompression with gradually decreasing inflammatory cord findings.
